# Predictive integration of gene functional similarity and co-expression defines treatment response of endothelial progenitor cells

**DOI:** 10.1186/1752-0509-5-46

**Published:** 2011-03-30

**Authors:** Francisco J Azuaje, Haiying Wang, Huiru Zheng, Frédérique Léonard, Magali Rolland-Turner, Lu Zhang, Yvan Devaux, Daniel R Wagner

**Affiliations:** 1 Laboratory of Cardiovascular Research, Centre de Recherche Public - Santé, L-1150, Luxembourg; 2 School of Computing and Mathematics, Computer Science Research Institute, University of Ulster, Newtownabbey, BT37 0QB, UK; 3 Division of Cardiology, Centre Hospitalier, L-1210, Luxembourg

## Abstract

**Background:**

Endothelial progenitor cells (EPCs) have been implicated in different processes crucial to vasculature repair, which may offer the basis for new therapeutic strategies in cardiovascular disease. Despite advances facilitated by functional genomics, there is a lack of systems-level understanding of treatment response mechanisms of EPCs. In this research we aimed to characterize the EPCs response to adenosine (Ado), a cardioprotective factor, based on the systems-level integration of gene expression data and prior functional knowledge. Specifically, we set out to identify novel biosignatures of Ado-treatment response in EPCs.

**Results:**

The predictive integration of gene expression data and standardized functional similarity information enabled us to identify new treatment response biosignatures. Gene expression data originated from Ado-treated and -untreated EPCs samples, and functional similarity was estimated with Gene Ontology (GO)-based similarity information. These information sources enabled us to implement and evaluate an integrated prediction approach based on the concept of *k*-nearest neighbours learning (*k*NN). The method can be executed by expert- and data-driven input queries to guide the search for biologically meaningful biosignatures. The resulting *integrated kNN *system identified new candidate EPC biosignatures that can offer high classification performance (areas under the operating characteristic curve > 0.8). We also showed that the proposed models can outperform those discovered by standard gene expression analysis. Furthermore, we report an initial independent *in vitro *experimental follow-up, which provides additional evidence of the potential validity of the top biosignature.

**Conclusion:**

Response to Ado treatment in EPCs can be accurately characterized with a new method based on the combination of gene co-expression data and GO-based similarity information. It also exploits the incorporation of human expert-driven queries as a strategy to guide the automated search for candidate biosignatures. The proposed biosignature improves the systems-level characterization of EPCs. The new integrative predictive modeling approach can also be applied to other phenotype characterization or biomarker discovery problems.

## Background

The impairment of the endothelium is a key factor driving the initiation and progression of different manifestations of heart disease [1]. Thus, the preservation or regeneration capability of the endothelial layer has crucial prognostic and therapeutic value [1,2]. An important vasculature repair mechanism consists of the activation of endothelial cell precursors, known as *endothelial progenitor cells *(EPCs). EPCs can differentiate into endothelial cells (ECs), which in turn may lead to regeneration of damaged tissue after a myocardial infarction [1,3]. EPCs have also been directly associated with different clinical stages of cardiovascular disease: from aging and atherosclerotic disease development, to acute myocardial infarction and heart failure [1]. EPCs have been suggested as promoters of vascular network regeneration in ischemic tissue in a paracrine fashion [3-5]. Additionally, adenosine (Ado) treatment has been investigated as a potential approach to promote vascular regeneration in ischemic tissue [6,7]. This motivates the formulation of new methods to characterize, molecularly and phenotypicaly, EPCs responses to Ado treatment. Moreover, it is still unclear how Ado can reconfigure the response transcriptional program of EPCs at a systems level.

Notwithstanding cumulative progress in the functional characterization of EPCs using genome-wide expression profiling [1,5], there is a lack of systems-level understanding of key interactions and processes controlling the response of EPCs to candidate therapeutic interventions. Recent systems biology advances have shown promise in the elucidation of potential biomarkers of phenotype and clinical outcomes, particularly in cancer research [8-11]. This has been done, for instance, by harnessing the predictive integration of gene expression data and other biological information available in publicly-funded, community-driven repositories [8,9,11,12]. Among such strategies, we and others have investigated the integration of gene expression data and standardized descriptions of the biological function of gene products, as well as different types of protein interaction data, to support the search for candidate prognostic biomarkers and therapeutic targets [13-15]. Specifically, researchers (including us) have demonstrated how measures of functional similarity based on Gene Ontology (GO) annotations can be applied as complementary predictive features to characterize gene expression profiles and protein-protein interactions [14,16,17].

Therefore, we reasoned that an integrative computational approach based on the combination of different biological data and information sources could offer new and deeper views of Ado-treatment response of EPCs in a holistic fashion. We also investigated the combination of hypothesis- and data-driven approaches to discovering biologically relevant molecular signatures of treatment response. We implemented these systems-driven, integrative strategies to improve understanding and characterization of EPCs in the context of Ado treatment.

### EPCs biosignature discovery strategy

**Figure 1:**
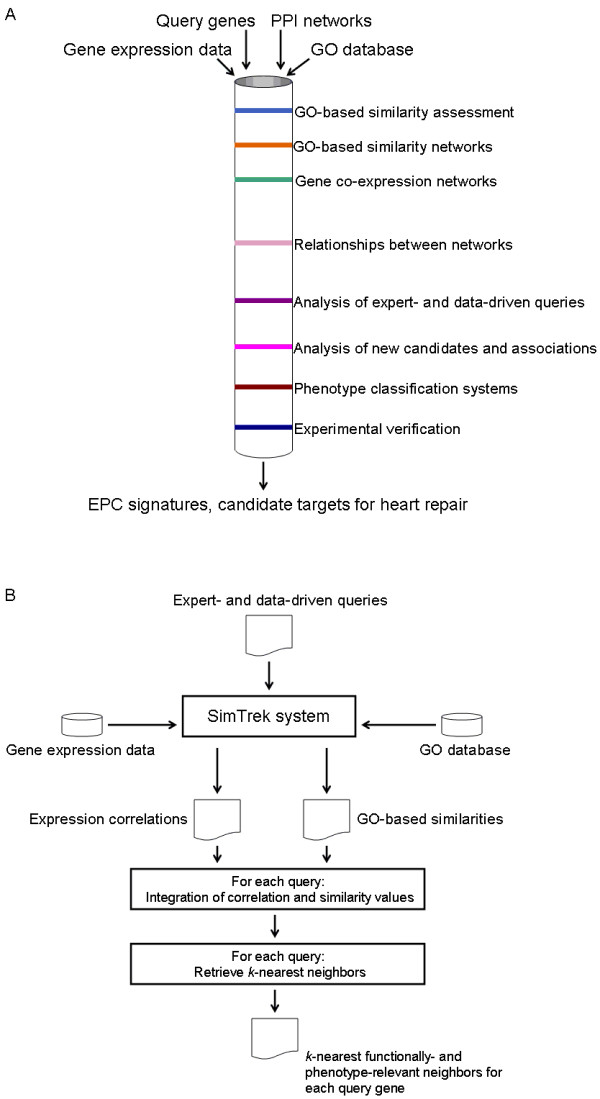
**Research framework and techniques implemented to characterize EPCs**. A. Schematic representation of the research workflow. B. Algorithmic description of the integrated *k*NN approach to identifying EPC biosignatures of treatment response.

The main inputs to our research pipeline were: microarray data from human EPCs, a comprehensive experimentally-validated network of human protein-protein interactions (PPI), human GO annotations, and different sets of research "queries" that represented initial guiding inputs to reduce the search space of potentially novel associations and biomarkers of EPCs activity (Figure 1A, and Methods). Note that the PPI is not required for implementing our proposed integrative method. The PPI network was used for implementing an alternative integrative approach to compare against our technique.

We investigated two types of queries: *Expert- *and *data-driven*. The former refers to genes of known relevance to EPCs identity or activity. The latter were derived from statistical analysis of the microarray data, and represented those genes that were highly differentially expressed between Ado-treated and -untreated EPCs.

GO-based functional similarity estimations and subsequent integrated analyses were implemented with the SimTrek system [18] (Methods). SimTrek computes the functional similarity between query genes and the other genes in the human genome. Functional similarity networks were then defined, in which nodes and edges represented gene products and their functional similarity levels respectively. We also built transcriptional association networks linking the query genes and all the genes measured in the microarray dataset. In this case the association between two genes was quantified as the gene expression correlation of the genes (Methods).

This was followed by examinations of quantitative relationships between the biological associations reported by the PPI, transcriptional and GO-based similarity networks. This enabled us to explore the predictive potential of these resources, as well as to detect novel biological associations relevant to the molecular characterization of EPCs. An important outcome of these tasks was the definition of a set of genes that can be used to characterize the differential response of EPCs to Ado treatment. To assess the predictive potential of this signature, we implemented different EPCs classification systems based on machine learning. Finally, as an initial step towards the independent validation of our findings, we performed independent protein expression profiling of one of the members of the EPCs signature. This indicated that the molecular activity of the top biosignature may also be reflected at the post-transcriptional level.

The combination of GO-based similarity and transcriptional association information between pairs of genes was at the centre of the EPCs signature discovery strategy investigated (Figure 1B). In this integrative data mining approach, gene expression correlations and GO-based similarity were computed between all the query genes and all those genes with gene expression data and GO annotations available. Thus, each query gene was linked to multiple genes through co-expression and GO-based similarity relationships. Based on the premise that these data sources can provide complementary functional information, the aggregation of co-expression and GO-based similarity values (the mean value) was used as a numerical score to represent the integrated functional relationship between a query and another gene. This search scheme provides a mechanism to retrieve and rank the most functionally similar genes to each query gene. Hereafter, this technique will be referred to as the integrated *k*-Nearest Neighbour (*k*NN) algorithm, with *k *representing the number of putative candidates that are retrieved as functionally related to a query gene. We hypothesized that query genes together with their most relevant *k*NNs may encode EPC signatures, which can provide a more accurate method to characterize the treatment response of these cells. Afterwards, to assess their potential predictive capacity, we applied the resulting biosignatures as inputs to automated EPCs classification systems.

## Results

We first investigated whether the integrated *k*NN method was capable to identify potentially relevant query-driven networks linked to EPCs treatment response. The expert-driven queries consisted of a set of 1 chemokine receptor and 6 cytokines with potential significant roles in EPCs development fate: CXCR4, CXCL2, CXCL5, CXCL12, CCL7, CCL2 and CCL23. These choices were based on preliminary experiments recently performed in our laboratory suggesting that Ado may regulate the expression of several members of the chemokines/chemokine receptors superfamily. In addition, the CXCR4/CXCL12 axis is known to be highly implicated in EPCs mobilization and recruitment to injury site [19-21]. In the cancer context, CXCL2 and CXCL5 have displayed pro-angiogenic properties [22]. Thus, this query set is relevant to determine whether Ado can have beneficial effects on EPCs recruitment or activation of their pro-angiognic properties through the modification of chemokine expression patterns.

**Figure 2:**
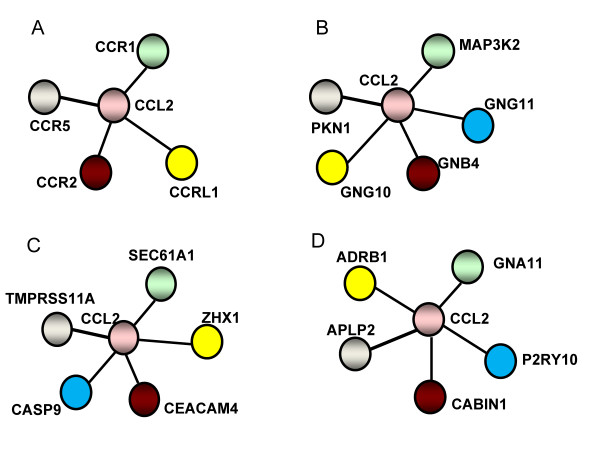
**Treatment response networks based on different data resources**. Examples of sub-networks defined by the 4-nearest neighbours to a query gene (CCL2). The nearest neighbours were identified on the PPI (A), GO-based similarity (B), co-expression (C) and the integrated *k*NN network spaces (D).

The data-driven queries consisted of 134 genes highly differential expressed between Ado-treated and -untreated EPC samples (6 vs. 6 samples, Significance Analysis of Microarrays, SAM, FDR < 0.001, fold-change = 1.7). Expert- and data-driven query sets did not share genes in common. Figure 2 illustrates examples of sub-networks defined by the different nearest neighbors to a query gene (CCL2) as seen on the PPI, co-expression, GO-based similarity and integrated *k*NN network spaces independently.

Surprisingly, the overlap between the sub-networks (neighbourhoods) using the PPI network and the integrated *k*NN method was almost null for all queries. This lack of overlap was estimated by comparing, for each query, the number of shared neighbourhoods detected by each method (null hypothesis: mean number of shared neighbours = 0, one-sample t-test, P = 0.98). This was consistently observed for different neighbourhood sizes (1≤ *k *≤ 20).

This suggests that, in principle, our integrated *k*NN methodology can offer complementary predictive capability for detecting candidate biosignatures of treatment response in EPCs. Moreover, we aimed to reveal novel functional relationships to characterize molecular response. Specifically, our objective was to answer the question: can we exploit this knowledge to improve the molecular classification of EPCs in response to Ado treatment?

### Treatment response biosignatures of EPCs

To discover biosignatures of treatment response, we built a variety of prediction models based on the genes identified by our integrated *k*NN method. The classification problem was to distinguish between Ado-treated from untreated samples. We compared its prediction performance against models derived from standard expression data analysis and information encoded in the PPI network. To facilitate comparisons across candidate biosignatures (the inputs to the prediction models) and to minimize the risk of model over-fitting, prediction models were built with Support Vector Machines (SVM) and classification performance was estimated with the Leave-One-Out Cross-Validation data sampling strategy (LOOCV). Areas under the receiver operating characteristic curve (AUC values) were used to summarize the classification performance of each model (Methods). Candidate biosignatures detected by our integrated *k*NN method were investigated for *k *= 1 to 20.

Also we encoded all candidate biosignatures using two model input representation schemes: 1. each model input represents an individual expression value corresponding to each selected gene, and 2. each model input encodes the integrated gene expression activity detected in the neighbourhood of a query gene, i.e., the expression values of all genes in a signature are averaged. The latter only applied to models based on the integrated *k*NN and PPI-based methods, with each input representing the mean expression value of the query and its neighbouring genes. Hereafter, we will refer to these input representation schemes as *individual gene *and *integrated gene neighbourhood *representations respectively. We also built multiple prediction models for different combinations of the most differentially expressed genes detected by SAM, and different number of (expert- and data-driven) query genes.

We will name different classification models with acronyms (and their combinations) that encode the characteristics of the models: EDQ (expert-driven queries), NN (our integrated nearest-neighbour technique), PPI (models based on neighbourhoods extracted from the PPI network) and DE (differential expression genes).

**Figure 3:**
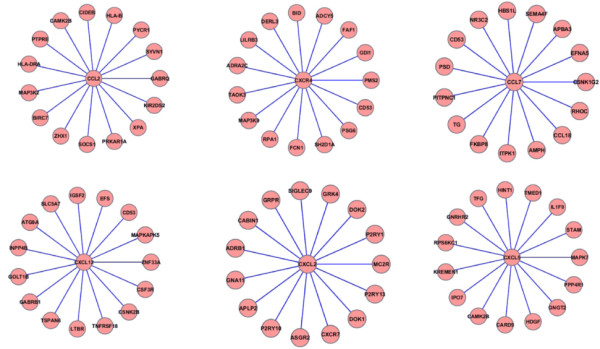
**Top network-based biosignature of treatment response in EPCs**. Gene composition and interactions of the biosignature "EDQ+15NN", which provided the basis for the best prediction model with 6 neighbourhood expression inputs.

**Figure 4:**
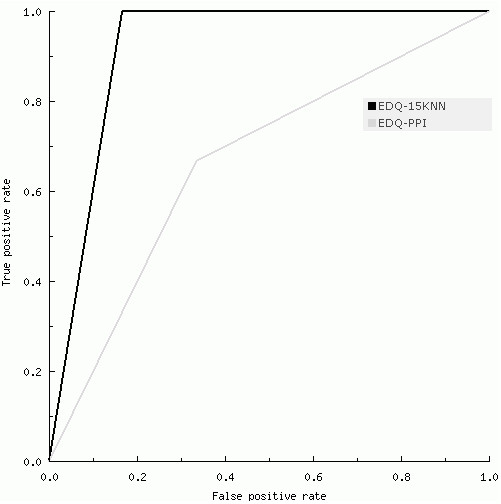
**ROC curves for representative prediction models**. Models compared: EDQ+15NN, EDQ and EDQ+PPI.

**Figure 5:**
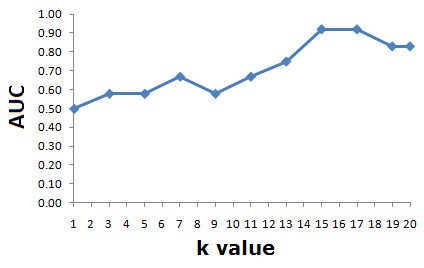
**The effect of *k *on the classification performance of our integrated method**. Models based on EDQ (6 expert-driven queries).

The most powerful prediction models were based on the integrated *k*NN technique, with integrated gene neighbourhood input representation (Table 1). A more detailed description of the gene composition of these biosignatures is given in Additional file 1. The top prediction model (AUC = 0.92) was derived from an integrated *k*NN model (*k *= 15) based on expert-driven queries: Models "EDQ+15NN" (Figure 3). In this model, the query genes were: CXCR4, CXCL2, CXCL5, CXCL12, CCL7 and CCL2. This top performance was followed by models based on highly differentially expressed genes (EFNA1, SH3BP5, PEA15 and B2 M, AUC = 0.75), and a model based on the integrated *k*NN approach using these genes as queries (AUC = 0.83, *k *= 4, and integrated gene neighbourhood representation). The best model based on individual gene input representation and expert-driven queries reported an AUC = 0.75 (Table 1). Models based on different query genes and their interacting partners in the PPI network reported poorer performance (maximum AUC = 0.67). Models based on the *k*NN method and the input representation scheme defined by individual genes exhibited poorer performance. In EDQ+15NN, GO-based similarity was estimated with BP terms. The performance of this model was reduced when using the MF hierarchy (AUC < 0.5). This may partly be explained by the relatively small number of query genes with high quality GO MF annotations. For example, among the expert queries this information was available only for CXCR4, CXCL12, and CCL2. Figure 4 displays the ROC curves for representative prediction models: EDQ+15NN, EDQ and EDQ+PPI. Figure 5 summarizes the effect of *k *on the classification performance of our integrated method based on EDQ (6 expert-driven queries).

**Table 1:** EPCs biosignatures of Ado-treatment response.

Name	Biosignature gene composition	BS	AUC	*k*
EDQ	Expert-driven queries: CXCR4, CXCL2, CXCL5, CXCL12, CCL7, CCL2, CCL23	7	0.75	*-*

EDQ+15NN	Expert-driven queries (CXCR4, CXCL2, CXCL5, CXCL12, CCL7, CCL2) together with their most functionally similar genes from integrated *k*NN strategy*	6	0.92	15

DE	Top-4 data-driven queries: EFNA1, SH3BP5, PEA15, B2M	4	0.75	-

DE+4NN	Top-4 data-driven queries together with their most functionally similar gene from integrated *k*NN strategy*	4	0.83	4

EDQ+PPI	Expert-driven queries (CXCR4, CXCL2, CXCL5, CXCL12, CCL7, CCL2) together with their interacting partners in the PPI network	6	0.67	-

Top classification performances (summarized with AUC values) obtained from different query-driven schemes and the integrated *k*NN. AUC values estimated through LOOCV and correspond to SVM classification models (see Methods). Prediction models based on biosignatures identified by standard statistical techniques or query-driven inputs only are also included (models indicated with " - "). "*": In EDQ+15NN, GO-based similarity in integrated *k*NN model was estimated with BP terms, in DE+4NN with MF terms. BS: Biosignature size defined as the number of inputs to prediction models based on two schemes: individual gene (EDQ and DE models) and integrated gene neighborhood input representations.

To further assess the potential predictive relevance of our approach, we implemented classification models based on network neighbourhoods retrieved by the STRING system [23]. We provided STRING with our expert-driven queries and retrieved interactions (neighbourhoods) for each of them. The average gene expression values of the neighbourhoods were applied as inputs to classification models as done with our integrated approach. We implemented analyses with up to 10 neighbouring genes/query and confidence scores > 0.9. Classification results were in general very poor (AUC < 0.5) for different combinations of queries and neighbourhoods. This may be partly explained by the low number of genes retrieved by STRING with gene expression data available in our dataset. This was the case of genes that could not be measured or did not meet fold-change requirements in our experiments. For example, the STRING-retrieved neighbourhood of query gene CXCL2 only included one gene, CXCL5, with expression measurements available. This supports the idea that our method is capable to detect relevant information that is not necessarily strongly bound to differential transcriptional behaviour alone.

We found genes that are shared by different neighbourhoods: CAMK2B (shared by CCL2 and CXCL5's neighbourhoods); CD53 (shared by CXCR4, CCL7, CXCL12); and HBS1L, NR3C2 and PSD (shared by CCL23 and CCL7). One may hypothesise that these overlapping genes could encode relevant biological information for treatment response prediction purposes. To test this assumption, we built different treatment response classifiers using their gene expression values as model inputs (i.e., independent input sets: CAMK2B; CD53; HBS1L, NR3C2 and PSD; and their combination). These models reported very low classification performance (AUC < 0.5). This emphasises the importance of applying an integrated and synergistic approach to prediction model design, as originally specified in our method.

To sum up, this systematic comparison of prediction models indicates that Ado-treatment response in EPCs can be accurately predicted by using models based on: query genes, our integrated *k*NN biosignature identification method and the integrated gene neighbourhood input representation scheme. Figure 3 shows the gene composition of the biosignature "EDQ+15NN", which provided the most powerful prediction model of treatment response. Although the signature is defined by 105 (7 × 15) genes, we stress that the inputs to the prediction model consisted of only 7 neighbourhood expression values. As a whole, this gene set encodes products that are strongly associated with intracellular signaling cascade (Fisher's exact test with Benjamini correction, P = 9E-6) and regulation of protein kinase cascade (P = 1.2E-3) as defined in the GO.

### Independent experimental follow-up of EPCs biosignature

**Figure 6:**
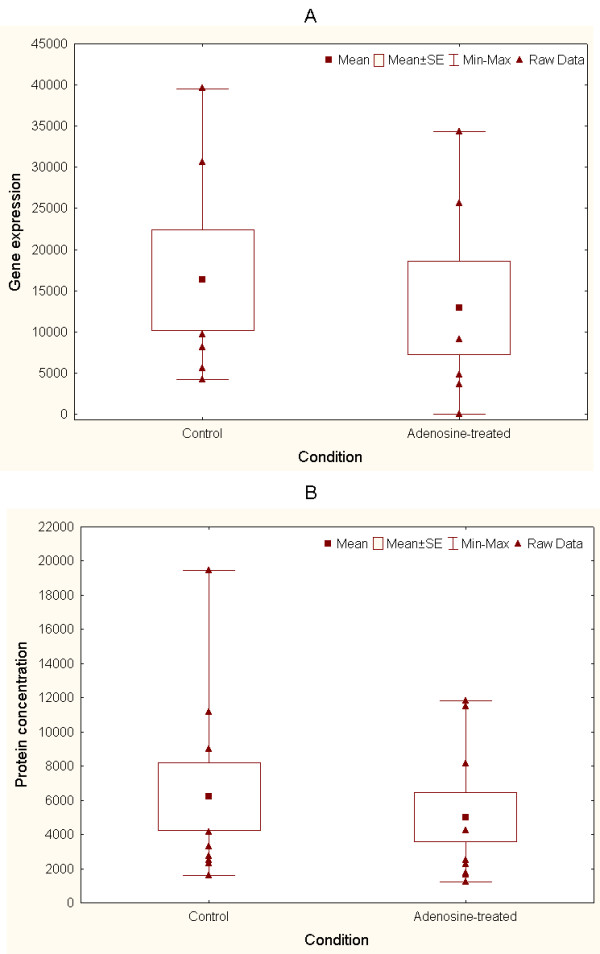
**Comparison of CCL18 gene and protein expression values in treated vs. control samples**. A: Gene expression values from Ado-treated and control samples (6 vs. 6 experiments, fold-change = 0.87, Mann-Whitney U test, P = 0.42). B: Protein concentration values from matched Ado-treated and control experiments (9 vs. 9 experiments), fold-change = 0.83, Wilcoxon matched-pairs test, P = 0.066)

As an initial step towards the independent validation of the predictive potential of the integrated *k*NN method, we measured protein expression levels encoded by one of the genes identified. This was done in 9 independent samples for the protein CCL18, which is known to be implicated in the regulation of immunological responses and inflammation, as well as over-expressed in several diseases [24]. More recently, CCL18 has been proposed as a potential diagnostic and prognostic parameter in patients with stable coronary artery disease [25]. In the microarray dataset, CCL18 displayed a reduction of expression in Ado-treated samples in relation to untreated samples, though not statistically detectable at P = 0.05 (6 treated vs. 6 control, intensity fold-change = 0.87) (Figure 6A). ELISA experiments on matched EPC samples (Figure 6B, and Methods) reported a detectable reduction of CCL18 protein concentration in Ado-treated samples (9 treated vs. 9 control, mean fold-change = 0.83, one-sample t-test: P = 2E-6, with fold-change = 0 as null hypothesis). A less statistically detectable difference was observed when comparing (control vs. Ado-treatment) raw concentration values (Wilcoxon matched-pairs test, P = 0.066).

These results indicate that: a. our integrated *k*NN method could detect a candidate biosignature that may be measurable at both gene and protein expression levels, and b. at least one of the members of this signature exhibits consistent differential responses at the transcriptional and post-transcriptional levels. This encourages the future implementation of independent evaluations of the predictive potential of the proposed biosignatures. Moreover, this suggests that different experimental measurement techniques, including qPCR and ELISA, may be applicable.

## Discussion and Conclusions

### New biological insights and potential clinical relevance

We showed that an integrated *k*NN method can identify candidate biosignatures of Ado-treatment response in EPCs. This biosignatures not only can improve the automated characterization of EPCs, but also can provide insights unobtainable by standard gene expression analysis or "guilt-by-association" methods in PPI networks. This is explained in part by the fact that the integrated *k*NN method combines predictive evidence, both functional and phenotype-specific, as encoded in GO annotations and whole-genome expression profiling experiments. At this point we consider both features as equally relevant, and we do not have evidence to suggest that a different scheme would provide better predictions. However, as part of future research, it would be important to investigate different feature weighing schemes. Our method also enables the incorporation of prior knowledge through the processing of expert-driven input queries. Additionally, we showed how (less biased) data-driven queries may also drive the discovery of predictive and biologically meaningful biosignatures.

A closer look at the genes identified by the integrated *k*NN method highlights additional insights about the biological relevance of the discovered top biosignature (Figure 3) to characterize treatment response of EPCs. Among the 15-nearest neighbours retrieved for each of the 7 expert-driven queries, more than 1/3 of them are annotated to GO terms implicated in cardiovascular development or disease, according to the Cardiovascular Gene Ontology initiative [26]. Other genes, such as FKBP8, a nearest neighbour to CCL7, is known to be involved in protein folding and trafficking [27], as well as mouse eye development [28]. The association between BIRC7 and neuroblastoma has been recently documented [29]. Interestingly, this signature included two known markers of susceptibility to congestive heart failure and beta-blocker response in congestive heart failure patients (ADRA2C and ADRB1, as annotated in the OMIM Disease database) [30]. RAF1, which was found in the "DE+4NN" biosignature, has been recently identified as a critical intracellular control point for inducing robust self-renewal of hematopoietic stem cells [31]. At the time of submitting this paper, the effect of Ado on CCL18 had not been reported in the literature. CCL18 is known to be elevated in inflammatory and pathological conditions [24]. Our results showed that Ado can decrease CCL18 expression, which is consistent with the anti-inflammatory and cardio-protective properties of Ado [6]. We did not find published evidence directly linking the members of this biosignature to Ado-treatment response in EPCs.

### Possible limitations

Interpretations of our findings and future investigations should take into account the following possible limiting factors. First, our study is constrained by the relatively small number of EPC samples analyzed. Despite this limitation, the integrative and knowledge-driven nature of our approach can aid in reducing the possibility of reporting spurious associations. Should we have focused on a purely data-driven approach (i.e., standard analysis of gene expression data), this problem would have represented a more critical influencing factor. Another key aspect to be considered is the relative small number of expert-driven queries analyzed, which entails that other potentially interesting biosignatures may have been missed in our investigation. However, to address the bias and incompleteness of such a hypothesis-driven approach, we also implemented analyses involving large-scale data-driven queries. Our findings showed that the expert-driven queries provide the basis for the most predictive biosignature, though non-redundant biosignatures with lower prediction performance can be obtained with the data-driven queries. Thus, our approach is capable to generate biologically meaningful predictions while minimizing the space of possible false positive associations. As new hypotheses emerge and more data are generated, future research can incorporate additional expert- and data-driven queries. Also we concede that a true independent validation of our approach will ideally consist of the measurement of all the biosignature members to test the classification models and input encoding schemes proposed here. The reported independent experimental follow-up for one of the top biosignature members at the protein expression level opens up a feasible alternative for future validations. Moreover, we are sharing our dataset through the Gene Expression Omnibus (GEO, accession number: GSE26744) [32], which may allow other researchers to conduct independent evaluations.

Future analyses could include comparisons of our technique versus PPI-based models in which the networks are assembled by other PPI integration strategies, such as the iRefWeb system [33]. To expand the comparison of our integrated method versus alternative solutions, the following systems are recommended as suitable options: STRING [23], FunCoup [34] and GeneMania [35]. The problem of biosignature multiplicity is a crucial challenge to achieve reproducible and clinically-relevant prognostic biomarkers. Such a multiplicity may be explained by different factors, among them, diversity of statistical techniques and data size constraints. Future evaluations of our integrated prediction approach and of our top biosignature could be examined with the aid of strategies that specifically consider reproducibility factors, such as those proposed by Boutros et al. [36] and Statnikov and Aliferis [37].

## Conclusions

We reported the predictive integration of: a. hypothesis and data-driven approaches, and b. gene expression and GO-based similarity information. We showed that such integration can enable the identification of networks of genes that may control the response to Ado-treatment in EPCs. In our integrated *k*NN approach, the definition of expert- and data-driven hypotheses represented a guiding principle for implementing a systematic search of candidate biosignatures. Thus, within a systems biology framework, the predictive integration of multiple functional and molecular information resources enabled the discovery of new biosignatures of treatment response in EPCs. This contributes to a more accurate characterization of EPCs and the understanding of their potential impact in clinical applications. Our integrated *k*NN approach may be suitable to other treatment response investigations, as well as other biomarker discovery applications.

## Methods

### Cell culture

EPCs were obtained from peripheral blood mononuclear cells (PBMC) of healthy patients by adhesion techniques as previously described [38]. All patients signed an informed consent. Briefly, PBMC were isolated from blood by ficoll density gradient centrifugation and then seeded onto human fribronectin (Sigma Aldrich, Bornem, Belgium) pre-coated plates in endothelial cell basal medium (EBM) supplemented with brain bovine extract, human endothelial growth factor, hydrocortisone, gentamicin, amphotericin B and 20% FCS (Lonza, Verviers, Belgium). After 3 days of culture, non-adherent cells were discarded and adherent cells were cultured for another 24 hours prior to treatment. Isolated EPCs were double positive for staining with lectin from Ulex europaeus (Sigma) and uptake of 1,1'-dioctadecyl -3,3,3',3'-tetramethyl-indocarbocyanine perchlorate (DiI-Ac-LDL). Flow cytometry characterization showed that isolated EPCs were CD133+/CD34+/CD45+/CD14+/vWF+/VEGFR2+/CD144-/CD105+. EPCs were treated with 10 μM Ado (Sigma) for either 6 h or 24 h for respectively micro array experiment or ELISA cytokine secretion assessment. 10 μM EHNA (erythro-9-(2-Hydroxy-3-nonyl) adenosine hydrochloride) was used as Ado deaminase inhibitor.

### Generation of microarray and protein expression data

For microarray experiments, the total RNA was extracted using TriReagent and the RNeasy mini kit according to manufacturer's instructions (Qiagen, Venlo, Netherlands). The RNA quality and quantity were evaluated with the Bioanalyzer and Nanodrop apparatus (Agilent). 1 μg total RNA was amplified using Amino Allyl MessageAmp kit (Ambion). 5 μg amino allyl-coupled RNA was labeled with Cy3 or Cy5 dyes (Amersham, Buckinghamshire, United Kingdom). Dye coupling yield >5% was a prerequisite for further analysis. 750 ng of labeled RNA was hybridized on 25,000 gene microarrays for 17 hours at 60°C. 4 arrays per sample were hybridized and scanned with the Genepix 4000B Scanner (Molecular Devices). Six independent experiments were performed. For protein expression assessment, cells were harvested and conditioned medium supplemented with protease inhibitors cocktail (Roche, Vilvoorde, Belgium) were stored at -80°C until use. Nine independent experiments were performed.

### Gene expression data analysis

Microarray data quantification and pre-processing was performed with the MAIA software [39] and intensity values were log-transformed. Gene expression values were standardized across experiments with mean = 0 and standard deviation = 1. The SAM tool was applied to identify differentially expressed genes, which then represented our set of data-driven queries. We focused on the most highly differentially expressed genes (fold-change = 1.7, FDR = 0.01). This dataset is available at the GEO [32], accession number: GSE26744.

### CCL18 ELISA assay

Concentration of CCL18 in conditioned medium was measured using the Human CCL18/PARC DuoSet ELISA (R&D Systems, Abingdon, United Kingdom) according manufacture's instructions.

### Generation of PPI network

The PPI network was assembled by aggregating experimentally validated human PPIs from the DIP [40], IntAct [41] and MINT [42] databases. These databases were chosen for their demonstrated interactome coverage, complementarity and low-error [40].

### GO-based similarity assessment

The estimation of gene-gene similarity using GO terms requires two main steps: 1. Calculation of the between-term similarity assigned to each gene, and 2. Aggregation of the between-term similarities to estimate the between-gene similarity. In this study GO terms were derived from human annotations downloaded from the GO database, and GO-based similarity was computed using MF and BP independently. We concentrated on non-IEA (non-Inferred from Electronic Annotation) term-gene associations. The estimation of between-term similarity was based on an information theory metric, Lin's semantic similarity measure [44], which has been previously investigated by authors of this study and others [14,16,17]. Between-term similarity was estimated based on the premise that the more information two terms share in common, the more similar they are, and that this can be quantified by looking at both the GO hierarchy structure and statistical information of gene-term associations [15,44]. Aggregation of between-terms similarities was done with the highest between-term similarity approach, which selectively aggregates maximum between-gene similarity values [18]. Given a pair of gene products, *g_i _*and *g*_j_, annotated to a set of GO terms *A_i _*and *A_j _*respectively, the GO-driven similarity, *SIM*(*g_i_*, *g_j_*), is calculated by aggregating maximum inter-set similarity values as follows:

These calculations were implemented with the SimTrek system [18] under the Cytoscape platform [45].

### Integrated kNN approach

The integrated *k*NN algorithm is summarized in Figure 1B. For each input query, its GO-based similarity and gene expression (Pearson) correlation values with the other genes measured in the microarray were computed. These values were normalized 0[1] prior to their combination. The correlation values (originally between -1 to 1) were transformed by applying the absolute value function (resulting values from 0 to 1). Their mean value was used to rank candidate genes in relation to each query. The *k*-most-similar genes were retrieved and defined the query's neighbourhood. We performed analyses for *k *= 1 to 20. This algorithm was implemented in an adapted version of the open-source SimTrek system [18].

### Treatment response prediction systems

We built different classification systems in which the inputs represented gene expression values or mean neighbourhood expression values. These schemes (introduced in Results) represented the individual gene and integrated gene neighbourhood input representation schemes respectively. We evaluated independent models to classify Ado-treated vs. -untreated samples based on inputs detected by SAM and the integrated *k*NN approach. Different combinations of individual gene and integrated gene neighbourhoods were investigated, including integrated *k*NN models with *k *= 1 to 20. The classification performance of the models was estimated using AUC values and LOOCV. SVM-based models were implemented based on their demonstrated classification capability and robustness [46]. To further reduce the possibility of data over-fitting, we concentrated on linear SVM models (John Platt's sequential minimal optimization algorithm, c = 100, exponent = 1).

### Statistical and bioinformatic tools

Microarray data pre-processing and differential expression analysis were conducted with MAIA [39] and SAM [47] respectively. The integrated *k*NN approach was implemented with the Java-based, Cytoscape-compatible SimTrek system [18]. Network visualization tasks were carried out with Cytoscape [45]. Other standard statistical analyses were done with the *Statistica *package [48]. Classification models were implemented with Weka [49]. GO term enrichment analysis and gene-disease association searches were done with the David system [50] and PubMed.

## List of abbreviations used

AUC: Area under the receiver operating characteristic curve; BP: GO biology process hierarchy; EPCs: Endothelial progenitor cells; GO: Gene Ontology; *k*NN: *k*-nearest neighbors; LOOCV: Leave-one-out cross-validation; MF: GO Molecular function hierarchy; SVM: Support vector machine.

## Authors' contributions

FA, MRT and YD conceived the study. FA and HW designed the integrated computational approach. HW and HZ performed computational experiments. FL and MRT performed in vitro experiments. FA, HY, HZ and LZ analyzed data. FA, MRT, YD and DW interpreted findings. FA prepared the manuscript assisted by the other authors. All the authors read and approved the manuscript.

## Supplementary Material

Additional file 1 **Gene composition of top biosignatures**. Table in PDF format describing top biosignatures. Click here for file
