# Supplementary material for: Predictive integration of gene functional similarity and co-expression defines treatment response of endothelial progenitor cells
Source: BMC Syst Biol. 2011 Mar 30;5:46. doi: 10.1186/1752-0509-5-46 (PMC3080295; doi:10.1186/1752-0509-5-46)
Supplement: Additional file 1 — Gene composition of top biosignatures. Table in PDF format describing top biosignatures. [file 1752-0509-5-46-S1.PDF]

Table S1. Gene composition of top biosignatures

Biosignature name	Biosignature gene composition
EDQ	CXCR4,CXCL2,CXCL5, CXCL12,CCL7,CCL2,CCL23
EDQ+15NN	ADCY5,ADRA2C,ADRB1,AKAP11,AMPH,APBA3,APLP2,ASGR2,ATG9A,BID,BIRC7,CABIN1,CAMK2B,CAMK2B,CARD9,CCL18,CD53,CIDEB,CSF3R,CSNK1G2,CSNK2B,CXCL13,CXCR7,DERL3,DOK1,DOK2,EFNA5,EFS,FAF1,FCN1,FKBP8,GABRB1,GABRQ,GDI1,GDI2,GNA11,GNGT2,GNRH2,GNRHR2,GOLT1B,GRK4,GRPR,HBS1L,HDGF,HINT1,HLA-B,HLA-DRA, IGSF2, IL1F9, INPP4B, IPO7, ITPK1,KCNIP3,KIR2DS2,KREMEN1,LILRB3,LTBR,MAP3K2,MAP3K9,MAPK7,MAPKAPK5,MC2R,NR3C2,P2RX1,P2RY1,P2RY10,P2RY13,PDE7A,PEX11B,PITPNC1,PMS2,PPP1R1B,PPP4R1,PRKAR1A,PSD,PSG6,PTPRE,PYCR1,RASGRP3,RHOC,RPA1,RPS6KC1,SCN1B,SEMA4F,SH2D1A,SIGLEC9,SLC5A7,SOCS1,STAM,STAMBP,SYVN1,TAOK3,TFG,TG,TMED1,TNFRSF18,TSPAN6,XPA,ZHX1,ZNF33A
DE	EFNA1, SH3BP5, PEA15, B2M
DE+4NN	EFNA1, EFNA2, AGRP, DIAPH1, C3, SH3BP5, KLRK1, SNX2, JAK3, UNC13D, PEA15, POLE3, AURKA, RAF1, SAT1, B2M, BCL2L11 ,THOC4, RAD17, TCEB2
EDQ+PPI	CXCR4, CD74, TLR2, MYH9, CCR5, CXCL2, IL8RB, DPP4, PIK3R5, CXCL5, IL8RB, IL8RA, CXCL12, DPP4, DPP8, DIP-400N, CCR4, CCR10, CCL7, CXCR3, FEZ1, CCRL1, CCR3, CCR5, CCR2, CCR1, AES, CCL2, CCRL1, CCR5, CCR2, CCR1
EDQ+15NN-v2	CCL2, MAP3K2, HLA-DRA, BIRC7, PTPRE, ZHX1, CAMK2B, SOCS1, CIDEB, PRKAR1A, HLA-B, XPA, PYCR1, KIR2DS2, SYVN1,GABRQ<br>CXCR4,ADCY5,PSG6,FAF1,CD53,GDI1,PMS2,TAOK3,ADRA2C,MAP3K9,LILRB3,RPA1,DERL3,FCN1,BID,SH2D1A<br>CXCL2,GNA11,ADRB1,APLP2,CABIN1,P2RY10,GRPR,ASGR2,SIGLEC9,CXCR7,GRK4,DOK1,DOK2,P2RY13,P2RY1,MC2R<br>CXCL5,KREMEN1,RPS6KC1,IPO7,GNRHR2,CAMK2B,TFG,CARD9,HINT1,HDGF,TMED1,GNGT2,IL1F9,PPP4R1,STAM,MAPK7<br>CXCL12,NPP4B,GABRB1,ATG9A,TSPAN6,SLC5A7,LTBR,IGSF2,TNFRSF18,EFS,CSNK2B,CD53,CSF3R,MAPKAPK5,ZNF33A,GOLT1B<br>CCL7,PITPNC1,PSD,TG,CD53,FKBP8,NR3C2,ITPK1,HBS1L,AMPH,SEMA4F,CCL18,APBA3,RHOC,EFNA5,CSNK1G2
